# Near-infrared fluorescence imaging of prostate cancer using heptamethine carbocyanine dyes

**DOI:** 10.3892/mmr.2014.2815

**Published:** 2014-10-29

**Authors:** JIANLIN YUAN, XIAOMIN YI, FEI YAN, FULI WANG, WEIJUN QIN, GUOJUN WU, XIAOJIAN YANG, CHEN SHAO, LELAND W.K. CHUNG

**Affiliations:** 1 Department of Urology, Xijing Hospital, Fourth Military Medical University, Xi’an, Shaanxi 710032, P.R. China; 2 Uro-Oncology Research Program, Department of Medicine, Cedars-Sinai Medical Center, Los Angeles, CA 90048, USA

**Keywords:** near-infrared fluorescence dye, fluorescence imaging, prostate cancer, circulating tumor cells, organic anion transporting peptides

## Abstract

Near-infrared fluorescence (NIRF) imaging is an attractive novel modality for the detection of cancer. A previous study defined two organic polymethine cyanine dyes as ideal NIRF probes, IR-783 and its derivative MHI-148, which have excellent optical characteristics, superior biocompatibility and cancer targeting abilities. To investigate the feasibility of NIRF dye-mediated prostate cancer imaging, dye uptake and subcellular co-localization were investigated in PC-3, DU-145 and LNCaP human prostate cancer cells and RWPE-1 normal prostate epithelial cells. Different organic anion transporting peptide (OATP) inhibitors were utilized to explore the potential role of the OATP subtype, including the nonspecific OATP inhibitor bromosulfophthalein, the OATP1 inhibitor 17β-estradiol, the selective OATP1B1 inhibitor rifampicin and the selective OATP1B3 inhibitor cholecystokinin octapeptide. NIRF dyes were also used for the simulated detection of circulating tumor cells and the rapid detection of prostate cancer in human prostate cancer tissues and prostate cancer xenografts in mouse models. The results revealed that the cancer-specific uptake of these organic dyes in prostate cancer cells occurred primarily via OATP1B3. A strong NIRF signal was detected in prostate cancer tissues, but not in normal tissues that were stained with IR-783. Prostate cancer cells were recognized with particular NIR fluorescence in isolated mononuclear cell mixtures. The results of the present study demonstrated that NIRF dye-mediated imaging is a feasible and practicable method for prostate cancer detection, although further investigative studies are required before clinical translation.

## Introduction

Prostate cancer (CaP) ranks first among the top ten most common types of cancer in males, and second highest as a cause of cancer-related mortalities. It was estimated that in the United States in 2014 there would be ~233,000 newly diagnosed patients with CaP and ~29,480 CaP-related mortalities (1). Although the majority of patients with localized low-risk CaP survive for a long time without any intervention, the remainder experience advanced disease with a high incidence of deterioration, recurrence and metastasis, all of which are associated with a poor prognosis and a higher risk of mortality (2). Currently, conventional imaging technologies are not sufficiently sensitive to detect small lesions or metastases in the early stage of the disease, providing little information for the identification of aggressive and indolent disease (3). Therefore, it is of great importance to develop cancer-specific molecular imaging techniques to improve CaP management.

Near-infrared fluorescence (NIRF) imaging is an attractive novel modality for cancer detection and the acquisition of real-time pathophysiological information. This imaging technique requires NIRF probes with an emission wavelength in the near-infrared region, similar to the contrast agents applied prior to positron emission tomography. There are a number of important requisites for these probes, including excellent optical characteristics, suitable biocompatibility and a cancer targeting ability (4). Two organic polymethine cyanine dyes have previously been described as ideal NIRF probes, IR-783 and its derivative MHI-148. These dyes accumulate selectively at tumor sites but not in normal tissues, possibly due to the differential expression of organic anion transporting peptides (OATPs) in cancer cells (5). OATPs are 12-transmembrane glycoproteins originating from the SLCO gene superfamily (6) that are expressed in several epithelial tissues throughout the body. In addition, OATP overexpression affects cancer development, including OATP1B3 in prostate cancer (7,8).

IR-783 and MHI-148 have great potential in the detection of malignancies without the requirement for additional conjugation with cancer-specific moieties, a method that has been widely used in the application of other NIRF probes (9–11). To further expand their clinical value in prostate cancer detection, the underlying mechanism and the feasibility of NIRF dye-mediated imaging was explored in different experimental settings, including both *in vivo* and *in vitro* studies. The aim of the present study was to investigate the feasibility of NIRF dye-mediated prostate cancer imaging, using IR-783 cyanine dyes. The dye uptake and subcellular co-localization in human prostate cancer cells PC-3, DU-145 and LNCaP and normal prostate epithelial cells RWPE-1 was tested.

## Materials and methods

### Chemical reagents

IR-783 cyanine dye was purchased from Sigma-Aldrich (St. Louis, MO, USA). MHI-148 was synthesized and purified as previously described (12). All dyes were prepared as stock solutions (1 mM) in dimethyl sulfoxide (DMSO; Sigma-Aldrich) and stored at 4°C in the dark. The dyes were diluted in serum free medium to an appropriate working solution and filtered through 0.2 μm filters prior to use.

### Cell lines and cell culture

PC-3, DU-145 and LNCaP human prostate cancer and RWPE-1 normal prostate epithelial cell lines were purchased from the American Type Culture Collection (ATCC, Manassas, VA, USA) and grown according to ATCC recommendations. Each of the recommended media (RPMI-1640 for LNCaP, F-12 Ham’s Kaighn’s modification medium for PC-3 and minimal essential medium for DU-145; Invitrogen Life Technologies, Carlsbad, CA, USA) contained 10% fetal bovine serum (Gibco-BRL, Carlsbad, CA, USA) and penicillin (100 IU/ml)/streptomycin (100 μg/ml) and the cells were cultured in a humidified atmosphere with 5% CO_2_ at 37°C.

### In vitro study of dye uptake in cultured cells

The cell staining procedures were undertaken as described previously (12). In brief, suspensions of PC-3, DU-145, LNCaP, and RWPE-1 (1×10^4^/well) cells were placed into four-chamber slides (Nalgen Nunc International, Penfield, New York, USA) and cultured for 24 h. Following the removal of the culture medium, working solutions of IR-783 or MHI-148 dyes (20 μM) were added. The slides were incubated at 37°C for 30 min and then washed twice with phosphate-buffered saline (PBS). The cells were counterstained using DAPI at 37°C for 10 min, followed by a two PBS washes and a 10-min fixation with 4% paraformaldehyde (Sigma-Aldrich). The slides were covered with glass coverslips using aqueous mounting medium (Sigma-Aldrich) and observed under a confocal laser microscope (OLYMPUS FV1000; Olympus, Tokyo, Japan) with an excitation wavelength of 633 nm and an emission wavelength of 670–810 nm (5).

Subcellular localization of the dyes in the prostate cancer cells was detected according to a previously established protocol (12). Briefly, commercially available probes, Mito Tracker Orange CMTMRos and Lyso Tracker Green DND-26 (Molecular Probes, Camarillo, CA, USA), were used to track cytoplasmic mitochondria and lysosomes. Following DAPI staining, the slides were placed in 500 nM CMTMRos for 30 min at 37°C followed by repeated rinsing. Subsequently, 200 nM DND-26 was added for 60 min at 37°C. Following repeated washes and mounting, the slides were observed under a confocal microscope (OLYMPUS FV1000; Olympus).. The emission/excitation wavelengths for CMTMRos were 504 nm/511 nm and for DND-26 were 554 nm/576 nm. Images captured in the same visual field in varying light conditions were merged for co-localization analysis of the NIRF cyanine dyes.

Prostate cancer cells were pre-incubated with different OATP inhibitors to determine the underlying mechanisms attributed to their specific uptake and accumulation of cyanine dyes. Nonspecific OATP inhibitor bromosulfophthalein (BSP, 250 μmol/l), OATP1 inhibitor rifampicin (20 μmol/l), selective OATP1B1 inhibitor 17β-estradiol (EST, 20 μmol/l), and selective OATP1B3 inhibitor cholecystokinin octapeptide (CCK-8, 20 μmol/l) were added to the prostate cancer cells for 5 min, which was followed by the previously mentioned staining procedures (13–15). The uptake and accumulation of the dyes with or without inhibitors was observed under a confocal microscope (OLYMPUS FV1000; Olympus). For comparative studies, flow cytometry was applied to determine the fluorescence intensity of each group. The prostate cancer cells (1×10^4^) were cultured in 6-well plates for 24 h, followed by staining with the NIRF dyes as previously described. Following a final PBS wash, the fluorescence of each tube was measured using a flow cytometer (FACS Aria; BD Biosciences), with excitation/emission wavelengths of 633 nm/780 nm for NIRF dye detection. The relative fluorescence intensity in each group was calculated as a percentage of the fluorescence intensity of the group without inhibitor application.

### Detection of prostate cancer cells in human blood samples

An experimental model was established to evaluate the feasibility of NIRF dye-mediated imaging of prostate cancer cells in human blood. Heparinized human blood samples were obtained from healthy volunteers following the approval of the Institutional Ethics Committee. Informed consent was obtained from all individuals. The single-cell suspension of prostate cancer cells was pre-labeled with DAPI, mixed into human blood (10–10^4^/ml) and NIRF dye (20 μmol/l) was added. The mixture was gently vortexed several times and the mononuclear and cancer cells were isolated via the gradient centrifugation method using Ficoll-Paque™ solution (GE Healthcare, Little Chalfont, United Kingdom) as previously described (16). The cells were then labeled with NIRF dyes as previously discussed (12). Subsequently, the mixture of normal and cancer cells was fixed and observed under a confocal microscope to identify the prostate cancer cells via the colored staining. The total number of prostate cancer cells in the blood was counted by flow cytometry.

### NIRF imaging of human prostate cancer tissues

Samples of human prostate cancer tissue and the adjacent noncancerous tissues were obtained with informed consent from five patients at Xijing Hospital to test the possibility of direct and selective NIRF dye staining in *ex vivo* settings. Retrieved tissues were stained with cyanine dyes for NIRF imaging using the IVIS Lumina II imaging station (Caliper Life Sciences, Hopkinton, MA, USA). The nonspecific IR-800 dye (Sigma) was used as a control. For confocal microscopy, the tissues were embedded in optimum cutting temperature medium (Sakura Finetek, Torrance, CA, USA). Frozen 10 μm sections were cut and stained with DAPI and the mounted sections were analyzed with a confocal microscope to observe the NIRF signals. For hematoxylin and eosin (H&E) staining, tissues were fixed in 4% paraformaldehyde and embedded in paraffin. Following H&E staining, the tissue sections (5 μm) cut from the paraffin blocks were examined and imaged by an expert pathologist (17) using an Olympus DP-72 digital camera (Olympus). Furthermore, following anesthesia with 2% isoflurane in 100% oxygen at a delivery rate of 1.5 l/min (18), slices of freshly harvested tumor specimens (1 mm × 2 mm × 2 mm) were implanted subcutaneously to the dorsolateral part of 4–6 weeks old athymic nude mice (n=3), which were obtained from the experimental animal center of the Fourth Military Medical University (Shaanxi, China) and had free access to food and water under a normal light/dark cycle. All animal experiments were conducted in accordance with the Animal Care and Use Committee Guidelines of the Fourth Military Medical University. The implanted mice were left for 24 h and then NIRF dyes were injected intraperitoneally at a dose of 0.375 mg/kg. During the following five days, mice bearing prostate cancer implants underwent NIRF imaging using the IVIS Lumina II imaging station.

### Evaluating NIRF imaging in mice models of prostate cancer

Human prostate cancer cells (1×10^6^) were implanted either subcutaneously, intraosseously or orthotopically into athymic nude mice (n=3, respectively) following the procedures previously reported (19). Once the tumors reached ~5–10 mm in diameter as assessed by macroscopic observation, palpation or X-ray, cyanine dyes were injected intraperitoneally at a dose of 0.375 mg/kg. Tumor-loaded mice were anesthetized 24 h post-NIRF dye injection. The whole body NIRF imaging of the mice was undertaken using an IVIS Lumina II imaging station. Following the imaging, mice underwent painless euthanasia by isoflurane overdose. The tissue distribution of the dyes within the mice was assessed. Frozen tissue sections together with paraffin-embedded tissue sections were obtained for immediate confocal imaging and H&E staining as described above.

### Data processing and statistics

Numerical data are expressed as the means ± the standard error of the mean (SEM). The statistical significance of data was determined by Student’s t test. SPSS 16.0 software (SPSS Inc., Chicago, IL, USA) was used for statistical analysis. P<0.05 was considered to indicate a statistically significant difference.

## Results

### Cultured prostate cancer cells selectively uptake and accumulate NIRF dyes

**Figure 1:**
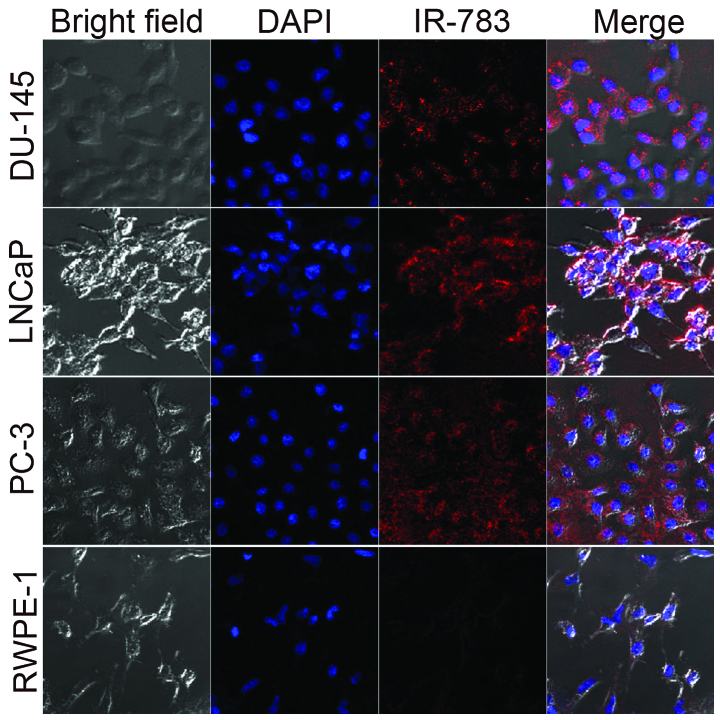
Selective dye uptake in DU-145, LNCaP, PC-3 prostate cancer cells. Only a weak near infrared fluorescence (red) was observed in RWPE-1 normal human prostate epithelial cells (magnification, ×200). The nuclei were stained with DAPI (blue).

PC-3, DU-145, LNCaP human prostate cancer cells and RWPE-1 normal human prostate epithelial cells were selected for *in vitro* studies to evaluate the NIRF dye uptake and retention by prostate cancer cells. Three images were captured of the same visual field of the prepared slides using confocal microscopy, including transparent, DAPI and NIRF imaging patterns (Fig. 1). Selective uptake and accumulation of NIRF dyes was observed in all prostate cancer cells. However, there was only a weak NIRF signal detected in RWPE-1 cells. These results confirm the findings of a previous study that these dyes have a unique cancer targeting ability (5).

**Figure 2:**
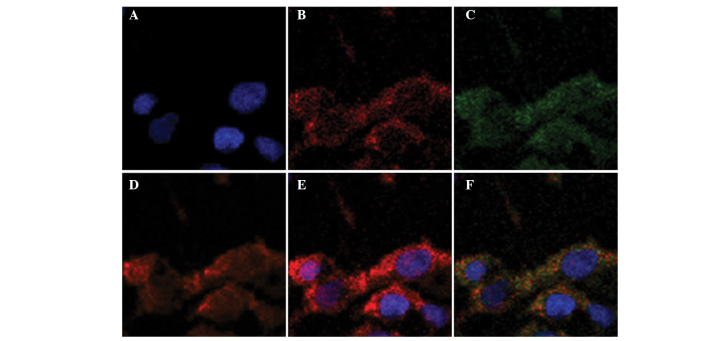
Preferential accumulation of near infrared dye in DU-145 prostate cancer cells. (A) DAPI (blue), (B) IR-783, (C) Lyso Tracker Green DND-26, (D) Mito Tracker Orange CMTMROS, (E) The superimposition of images A, B and D; (F) The superimposition of image A, B and C (magnification, ×400).

Previous studies have demonstrated the preferential retention of NIRF dyes in the lysosomes and mitochondria of the ARCaPM cell line, a highly metastatic subclone of ARCaP cell line derived from the ascitic fluid of a patient with prostate cancer (12). The present study investigated subcellular co-localization of NIRF dyes in three commonly used prostate cancer cell lines, PC-3, DU-145 and LNCaP. Merged images revealed a large overlap of cytoplasmic staining among the NIRF dyes in the lysosomes and mitochondria, indicating that a substantial portion of these dyes accumulate in the lysosomes and mitochondria of a number of prostate cancer cells (Fig. 2).

**Figure 3:**
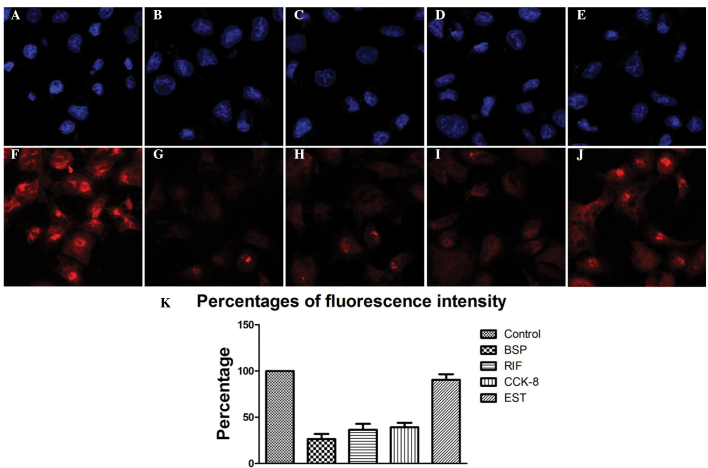
PC-3 prostate cancer cells were treated with different organic anion transporting peptide (OATP) inhibitors followed by staining with DAPI (blue) or IR-783 (red). (A, F) Control group with no treatment, (B, G) nonspecific OATP inhibitor bromosulfophthalein (BSP) group, (C, H) OATP1 inhibitor rifampicin (RIF) group, (D, I) selective OATP1B3 inhibitor cholecystokinin octapeptide (CCK-8) group, (E, J) selective OATP1B1 inhibitor 17β-estradiol (EST) group (magnification, ×200). (K) Fluorescence intensity in each group evaluated by flow cytometry.

Previous studies have demonstrated that the inhibition of OATPs greatly affects the uptake of NIRF dyes, however, the explicit mechanism remains elusive (11,20). Elevated expression levels of OATP1B3 have been observed in prostate cancer, therefore, the current study employed four types of OATP inhibitor to test whether this subfamily of OATPs was involved in NIRF uptake in prostate cancer cells (6,21,22). There were remarkable disparities in the uptake and accumulation of these dyes when different inhibitors were applied (Fig. 3). Nonspecific OATP inhibitor BSP induced the greatest impact on the NIRF signal, as revealed by the reduction in fluorescence intensity (26.4%±5.7%), compared with that of the control. The OATP1 inhibitor rifampicin (RIF) strongly diminished the fluorescence intensity (36.5±6.4%) compared with that in the control. Similarly, the selective OATP1B3 inhibitor CCK-8 significantly reduced the NIRF signal (39.2%±4.8%) compared with that of the control, although to a lesser extent than BSP or RIF. However, the selective OATP1B1 inhibitor EST had a minimal effect on the intensity of the fluorescence signal compared with that of the control group (Fig. 3). These results indicate that the selective uptake of NIRF dyes relies primarily on the transporting functions of OATP1B3.

### NIRF dye mediates the detection of circulating tumor cells in human blood samples

**Figure 4:**
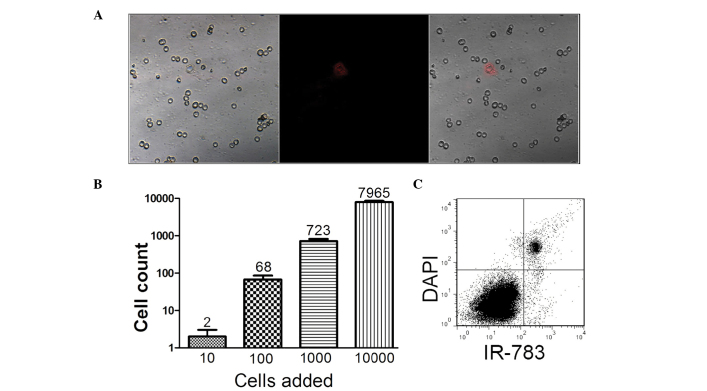
Near-infrared fluorescence (NIRF) dye-mediated imaging of prostate cancer cells in human blood. (A) The prostate cancer cells were detected by near infrared fluorescence (red) in human blood spiked with 10 cells (magnification, ×400). (B) The number of prostate cancer cells was counted by flow cytometry. (C) Results of flow cytometry revealed an apparent population of DAPI+/IR-783+ cells in human blood spiked with prostate cancer cells (104/ml).

Prostate cancer cells have the potential to migrate to distant organs via the circulatory system (23–25). Fluorescent probes with excitation/emission wavelengths in the visible region have been tested for the feasibility and reliability of detecting circulating tumor cells (CTC) in preclinical studies (16). An experimental model mimicking the detection procedure has previously been established to verify whether these NIRF dyes may be exploited for CTC detection (5). In the current study, blood samples were spiked with different numbers of prostate cancer cells (10–10^4^/ml). It was revealed that prostate cancer cells could be recognized with particular NIR fluorescence in isolated mononuclear cell mixtures, even at concentrations as low as 10 cells/ml in the blood (Fig. 4). Additionally, the results of the flow cytometric analysis support the viability of NIRF dye application in the detection of CTC in prostate cancer.

### NIRF imaging of human prostate cancer tissues using cyanine dyes

**Figure 5:**
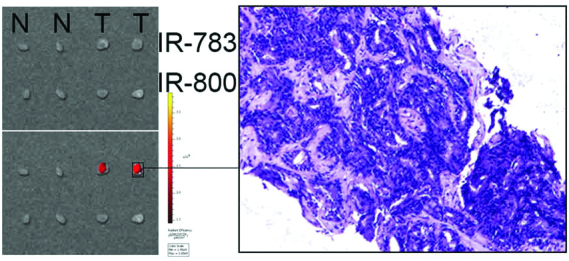
Near-infrared fluorescence (NIRF) imaging of prostate cancer tissues using IR-783. IR-800 failed to image tumors. Prostate cancer was confirmed by hematoxylin and eosin staining (magnification, ×200).

NIRF imaging was performed on human prostate cancer tissues, with the aim of determining if IR-783-mediated NIRF imaging remains effective at detecting prostate cancer cells in surgical samples. Samples of human prostate cancer tissues and the adjacent normal tissues were stained using IR-783 or IR-800. A strong NIRF signal was detected in the prostate cancer tissues but not in the normal tissues of the IR-783 group (Fig. 5). This result was confirmed by confocal microscopy and H&E staining. However, no difference was found in the IR-800 group, possibly owing to its lower stability and binding specificity to cancer cells. In addition, slices (1 mm × 2mm × 2mm) of prostate cancer tissue that were implanted subcutaneously into mice were detected by IR783, demonstrating the potential of IR-783-mediated NIRF imaging for clinical application.

### NIRF imaging of prostate cancer xenografts in mice models using cyanine dyes

**Figure 6:**
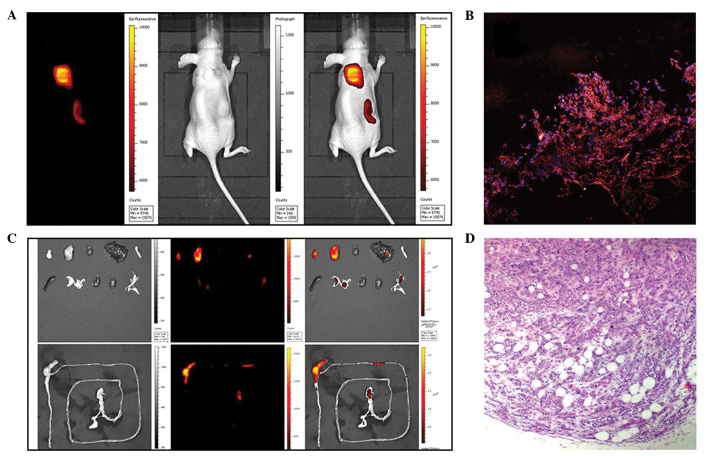
Near-infrared fluorescence (NIRF) imaging of prostate cancer xenografts in a subcutaneous mice model using IR-783. (A) Representative *in vivo* near infrared fluorescence images of subcutaneous prostate cancer (from left to right: NIRF image, bright light image and superimposition). Two clear tumors were identified. (B) Frozen sections of the retrieved xenografts were stained with DAPI and observed under a confocal microscope (magnification, ×200). (C) Bio-distribution of IR-783 in the organs of nude mice bearing prostate cancer. The top row of images show the following from upper left to lower right: tumor, tumor, heart, liver, pancreas, spleen, lung, kidney, kidney, bladder. Bottom row: the whole intestine. (D) Tumors were confirmed by hematoxylin and eosin staining (magnification, ×200).

Subcutaneous, intraosseous and orthotopical models of prostate cancer using athymic nude mice were established to validate the possible *in vivo* applications of NIRF imaging for the detection and observation of prostate cancer cells. NIRF dyes were administered and, 24 h later, high signal to background ratios were observed between the xenografts and the mouse models (Fig. 6). Bio-distribution analysis indicated that the metabolism of the NIRF dye was primarily through the excretions of bile, urine and feces. Furthermore, the NIRF signal from the prostate cancer cells remained detectable for >1 week. In sections of the xenografts retrieved from the sacrificed mice, the presence of prostate cancer cells and cancer specific uptake of NIRF dyes was confirmed.

## Discussion

Rapid progress has been made in the development of luminescent nanoparticles, and a number of them have been evaluated as potential contrast agents or delivery vehicles for molecular imaging, owing to their abilities of fast screening and early detection of cancer, which provide invaluable guidance in cancer therapy. However, conventional dyes are susceptible to photobleaching and rarely achieve a sufficient target-to-background ratio for clinical use, particularly due to the pharmacokinetics and potential toxicity associated with their concentration, surface coating, and chemical composition. NIRF dyes have received increasing attention in recent years for diagnostic imaging using near infrared light radiation, which penetrates up to 10 cm deep in certain tissues (20,26,27). The majority of NIRF dyes lack a specific targeting property, limiting their biomedical applications (28). In probe design using these dyes, a crucial targeting element has to be considered. Frequently used targeting moieties include antibodies, peptides, proteins, aptamers, and small receptor ligands (29,30). IR-783 and MHI-148 are two novel heptamethine indocyanine dyes that have been identified that preferentially accumulate in cancer tissues, hence displaying great advantages over the more commonly used fluorescent dyes with bioimaging applications (12). These NIRF dyes display dual imaging and targeting abilities in addition to a very low cytotoxicity.

The underlying mechanism of NIRF dye uptake remains elusive, although the contribution of sodium independent OATPs in dye uptake has previously been determined (31). In the present study, the roles of OATPs in prostate cancer detection were assessed through *in vivo* and *in vitro* experiments and the contribution of OATP subtype OATP1B3 was evaluated. OATP1B3 has previously been identified as an aberrantly expressed transporter in prostate cancer and has additionally been implicated in the progression of prostate cancer (8,22). The results of the current study indicate that OATP1B3 may be the predominant transporter involved in the dye uptake. These results are in good agreement with previous descriptions concerning the role of OATP1B3 in prostate cancer (14,32).

Cell imaging techniques for cancer diagnosis are simple, cost-effective, and relatively sensitive techniques that rely on the use of reporter genes and fluorescent dyes (33). The threshold sensitivity of photoacoustic flow cytometry is estimated to be as high as one cancer cell in a background of 107 normal blood cells (34). However, a fluorescence-based approach is only suitable for short-term labeling applications, while NIRF dyes allow for long-term tracking strategies with a satisfactory accuracy for diagnosis (5). In the current study, the labeling efficiency of NIRF dyes in all three types of prostate cancer cells was found to be satisfactory. Utilizing the tumor cell-specific behavior of these two dyes allowed for the detection of an accurate quantification of live CTCs in prostate cancer patients (23). Additionally, their preferential accumulation within prostate cancer xenografts allowed for noninvasive monitoring of uptake kinetics, tumor growth and therapeutic outcome.

NIRF imaging is a sensitive method of tagging a target of interest and is reliable for the noninvasive imaging of the microscopic and macroscopic levels, however, detailed anatomical information cannot be obtained. Multifunctional NIRF probes, which combine NIRF dyes with imaging modalities that provide anatomical information, including MRI, CT, PET, SPECT and photoacoustic imaging (PAI), are the next aim of the relatively novel rising field of cancer-targeting NIRF imaging technology (35–37). Whilst multifunctional NIRF probes are still far away from clinical application, it is anticipated that NIRF imaging technology will be unquestionably an integral part of biomedical research in the near future.

## References

[1] Siegel R, Ma J, Zou Z, Jemal A (2014). Cancer statistics, 2014. CA Cancer J Clin.

[2] Cai QY, Yu P, Besch-Williford C (2013). Near-infrared fluorescence imaging of gastrin releasing peptide receptor targeting in prostate cancer lymph node metastases. Prostate.

[3] Osborne JR, Akhtar NH, Vallabhajosula S, Anand A, Deh K, Tagawa ST (2013). Prostate-specific membrane antigen-based imaging. Urol Oncol.

[4] Yi X, Wang F, Qin W, Yang X, Yuan J (2014). Near-infrared fluorescent probes in cancer imaging and therapy: an emerging field. Int J Nanomedicine.

[5] Yang X, Shao C, Wang R (2013). Optical imaging of kidney cancer with novel near infrared heptamethine carbocyanine fluorescent dyes. Journal Urol.

[6] Svoboda M, Riha J, Wlcek K, Jaeger W, Thalhammer T (2011). Organic anion transporting polypeptides (OATPs): regulation of expression and function. Curr Drug Metab.

[7] Obaidat A, Roth M, Hagenbuch B (2012). The expression and function of organic anion transporting polypeptides in normal tissues and in cancer. Annu Rev Pharmacol Toxicol.

[8] Hamada A, Sissung T, Price DK (2008). Effect of SLCO1B3 haplotype on testosterone transport and clinical outcome in caucasian patients with androgen-independent prostatic cancer. Clin Cancer Res.

[9] Shimizu Y, Temma T, Hara I (2014). Micelle-based activatable probe for in vivo near-infrared optical imaging of cancer biomolecules. Nanomedicine.

[10] Chen Q, Wang C, Cheng L, He W, Cheng Z, Liu Z (2014). Protein modified upconversion nanoparticles for imaging-guided combined photothermal and photodynamic therapy. Biomaterials.

[11] Zhang E, Luo S, Tan X, Shi C (2014). Mechanistic study of IR-780 dye as a potential tumor targeting and drug delivery agent. Biomaterials.

[12] Yang X, Shi C, Tong R (2010). Near IR heptamethine cyanine dye-mediated cancer imaging. Clin Cancer Research.

[13] Ismair MG, Stieger B, Cattori V (2001). Hepatic uptake of cholecystokinin octapeptide by organic anion-transporting polypeptides OATP4 and OATP8 of rat and human liver. Gastroenterology.

[14] Karlgren M, Vildhede A, Norinder U (2012). Classification of inhibitors of hepatic organic anion transporting polypeptides (OATPs): influence of protein expression on drug-drug interactions. J Med Chem.

[15] Gui C, Wahlgren B, Lushington GH, Hagenbuch B (2009). Identification, Ki determination and CoMFA analysis of nuclear receptor ligands as competitive inhibitors of OATP1B1-mediated estradiol-17beta-glucuronide transport. Pharmacol Res.

[16] He W, Kularatne SA, Kalli KR (2008). Quantitation of circulating tumor cells in blood samples from ovarian and prostate cancer patients using tumor-specific fluorescent ligands. International J Cancer.

[17] Yi X, Zhang G, Yuan J (2013). Renoprotective role of fenoldopam pretreatment through hypoxia-inducible factor-1alpha and heme oxygenase-1 expressions in rat kidney transplantation. Transplant Proc.

[18] Valentim AM, Alves HC, Olsson IA, Antunes LM (2008). The effects of depth of isoflurane anesthesia on the performance of mice in a simple spatial learning task. J Am Assoc Lab Anim Sci.

[19] Wu TT, Sikes RA, Cui Q (1998). Establishing human prostate cancer cell xenografts in bone: induction of osteoblastic reaction by prostate-specific antigen-producing tumors in athymic and SCID/bg mice using LNCaP and lineage-derived metastatic sublines. Int J Cancer.

[20] Yuan A, Wu J, Tang X, Zhao L, Xu F, Hu Y (2013). Application of near-infrared dyes for tumor imaging, photothermal, and photodynamic therapies. J Pharm Sci.

[21] Buxhofer-Ausch V, Secky L, Wlcek K (2013). Tumor-specific expression of organic anion-transporting polypeptides: transporters as novel targets for cancer therapy. J Drug Deliv.

[22] Wright JL, Kwon EM, Ostrander EA (2011). Expression of SLCO transport genes in castration-resistant prostate cancer and impact of genetic variation in SLCO1B3 and SLCO2B1 on prostate cancer outcomes. Cancer Epidemiol Biomarkers Prev.

[23] Shao C, Liao CP, Hu P (2014). Detection of live circulating tumor cells by a class of near-infrared heptamethine carbocyanine dyes in patients with localized and metastatic prostate cancer. PloS On.

[24] Tjensvoll K, Nordgård O, Smaaland R (2014). Circulating tumor cells in pancreatic cancer patients: Methods of detection and clinical implications. Int J Cancer.

[25] Thalgott M, Rack B, Maurer T (2013). Detection of circulating tumor cells in different stages of prostate cancer. J Cancer Res Clin Oncol.

[26] Hellebust A, Richards-Kortum R (2012). Advances in molecular imaging: targeted optical contrast agents for cancer diagnostics. Nanomedicine (Lond).

[27] Fomina N, McFearin CL, Sermsakdi M, Morachis JM, Almutairi A (2011). Low power, biologically benign NIR light triggers polymer disassembly. Macromolecules.

[28] Muselaers CH, Stillebroer AB, Rijpkema M (2014). Optical imaging of renal cell carcinoma with anti-carbonic anhydrase IX monoclonal antibody girentuximab. J Nucl Med.

[29] Cheng K, Cheng Z (2012). Near infrared receptor-targeted nanoprobes for early diagnosis of cancers. Curr Med Chem.

[30] Bai M, Bornhop DJ (2012). Recent advances in receptor-targeted fluorescent probes for in vivo cancer imaging. Curr Med Chem.

[31] Letschert K, Faulstich H, Keller D, Keppler D (2006). Molecular characterization and inhibition of amanitin uptake into human hepatocytes. Toxicol Sci.

[32] Pressler H, Sissung TM, Venzon D, Price DK, Figg WD (2011). Expression of OATP family members in hormone-related cancers: potential markers of progression. PloS One.

[33] Shan L (2004). Near-infrared fluorescence 1,1-dioctadecyl-3,3,3,3- tetramethylindotricarbocyanine iodide (DiR)-labeled macrophages for cell imaging. Molecular Imaging and Contrast Agent Database (MICAD).

[34] Zharov VP, Galanzha EI, Shashkov EV, Khlebtsov NG, Tuchin VV (2006). In vivo photoacoustic flow cytometry for monitoring of circulating single cancer cells and contrast agents. Opt Lett.

[35] Qiao XF, Zhou JC, Xiao JW, Wang YF, Sun LD, Yan CH (2012). Triple-functional core-shell structured upconversion luminescent nanoparticles covalently grafted with photosensitizer for luminescent, magnetic resonance imaging and photodynamic therapy in vitro. Nanoscale.

[36] Kim JS, Kim YH, Kim JH (2012). Development and in vivo imaging of a PET/MRI nanoprobe with enhanced NIR fluorescence by dye encapsulation. Nanomedicine (Lond).

[37] Guo Y, Yuan H, Cho H (2013). High efficiency diffusion molecular retention tumor targeting. PloS One.

